# Ethical considerations of worksite health promotion: an exploration of stakeholders’ views

**DOI:** 10.1186/1471-2458-14-458

**Published:** 2014-05-16

**Authors:** Jantien van Berkel, Agnes Meershoek, Rien MJPA Janssens, Cécile RL Boot, Karin I Proper, Allard J van der Beek

**Affiliations:** 1 Department of Public and Occupational Health - EMGO Institute for Health and Care Research, VU University Medical Center, Amsterdam, The Netherlands; 2 Body@Work, Research Center on Physical Activity, Work and Health, TNO-VU University Medical Centre, Amsterdam, The Netherlands; 3 Department of Health, Ethics and Society, University Maastricht, Maastricht, The Netherlands; 4 VU University medical center, Department of Medical Humanities, EMGO Institute for Health and Care Research, Amsterdam, The Netherlands

**Keywords:** Worksite health promotion, Ethics, Focus group discussion, Face-to-face interviews, Stakeholder analysis

## Abstract

**Background:**

Developing, implementing and evaluating worksite health promotion requires dealing with all stakeholders involved, such as employers, employees, occupational physicians, insurance companies, providers, labour unions and research and knowledge institutes. Although worksite health promotion is becoming more common, empirical research on ethical considerations of worksite health promotion is scarce.

**Methods:**

We explored the views of stakeholders involved in worksite health promotion in focus group discussions and we described the ethical considerations that result from differences between these views. The focus group discussions were organised per stakeholder group. Data were analysed according to the constant comparison method.

**Results:**

Our analyses show that although the definition of occupational health is the same for all stakeholders, namely ‘being able to perform your job’, there seem to be important differences in the views on what constitutes a risk factor to occupational health. According to the employees, risk factors to occupational health are prevailingly job-related. Labour unions agree with them, but other stakeholders, including the employer, particularly see employee-related issues such as lifestyle behaviour as risk factors to occupational health. The difference in definition of occupational health risk factors translates into the same categorisation of worksite health promotion; employee-related activities and work-related activities. The difference in conceptualisation of occupational health risk factors and worksite health promotion resonates in the way stakeholders understand ‘responsibility’ for lifestyle behaviour. Even though all stakeholders agree on whose responsibility lifestyle behaviour is, namely that of the employee, the meaning of ‘responsibility’ differs between employees, and employers. For employees, responsibility means autonomy, while for employers and other stakeholders, responsibility equals duty. This difference may in turn contribute to ambivalent relationships between stakeholders.

**Conclusion:**

All stakeholders, including employees, should be given a voice in developing, implementing and evaluating worksite health promotion. Moreover, since stakeholders agree on lifestyle being the responsibility of the employee, but disagree on what this responsibility means (duty versus autonomy), it is of utmost importance to examine the discourse of stakeholders. This way, ambivalence in relationships between stakeholders could be prevented.

## Background

### Ethical considerations of worksite health promotion

Worksite health promotion has become more common in Western countries over the past years. A paradigm shift from occupational health to employees’ health contributed to this phenomenon [1]. Traditionally, the focus within the occupational setting used to be on reducing the harm of work (i.e. health protection), but nowadays, there are also more initiatives aimed at the promotion of health, e.g. lifestyle interventions [1]. Worksite health promotion is expected to occur even more often in the near future [2]. In addition, the WHO [2] has indicated the worksite is one of the priority settings for health promotion in the 21^st^ century. The setting and social network of the worksite is considered to lead to a potential large reach of individuals, which results in high expectations of health promotion in the occupational setting [2,3].

The worksite seems to be considered as a rather trouble-free or even favourable domain for health promotion. However, the suitability of the worksite for health promotion can be disputed [4,5]. The worksite is, in fact, not primarily intended for promoting health, but for working and for earning a salary [5]. A complicating factor of worksite health promotion is the relationship between the employer and the employee. Worksite health promotion assumes a shared interest in employees’ health, but employees have a dependency relationship with their employer; the employee depends on the employer with regard to aspects such as income.

### Exploring ethical considerations of worksite health promotion in literature

Research on worksite health promotion is characterised by ‘its focus on individual outcome measures, such as cost per employee, behaviour change or disease incidence’ [6], p77. This can be considered *a narrow definition of occupational health* and it results in a research agenda that focuses on employees as research subjects [6]. Employees’ health needs and their perspective on occupational health -which could be considered part of *a broad definition of occupational health-,* are not taken into account. Although the current research agenda is valuable in itself, it also has limitations due to the top-down approach of occupational health. Consequently, research on ethical considerations of worksite health promotion is scarce.

An example of an ethical consideration of worksite health promotion, found in literature, is *conflicting loyalties*. To whom does the professional promoting occupational health owe their allegiance and loyalty; to the employer, to the employee or both? This issue poses possible moral dilemmas when organizational goals and agenda are in conflict with the health needs and goals of the employees [6,7]. Another consideration is the risk of *blaming the victim*, especially on lifestyle-related topics. The tendency of most efforts in the worksite is to focus on the individual rather than on the nature of work and the organization itself. This might be explained by the ‘Just world hypothesis’ [8], a theory from social psychology, according to which we tend to view the world as ‘just’: people get what they deserve. Responsibility for health and illness is assigned exclusively to the individual employee. This would imply that worksite health promotion contributes to the individualization of organisational or collective problems and, as such, it may have an eroding impact on solidarity. Yet, individual behaviour is only one factor among others.

Health and illness are complex, multifactorial matters [6,7,9]. Related to this hypothesis is the more general concern that health promotion leads to *medicalization*[10]. Health promotion is said to contribute to tendencies in modern society where more and more aspects of everyday life are articulated in terms of ‘health’ and ‘illness’ [11,12]. Furthermore a side effect of such tendencies may be that increased knowledge of risk factors erodes feelings of confidence in one’s own health and instead increases feelings of insecurity. Another consideration is on *voluntariness*. Participation in worksite health promotion is presented as a free choice, but autonomy may be compromised because of the aforementioned dependency employer-employee relationship and peer pressure [7,13,14]. A last consideration is posed by *unintended consequences*. For example, worksite health promotion programs aimed at screening for health risk factors, might contribute to job discrimination, although well intended by the professionals [7].

### Stakeholders

As the exploration of ethical considerations of worksite health promotion in literature demonstrates, there are different stakes or interests in worksite health promotion. Health is not the only value involved, also autonomy, having work and equal chances at work play a role for example. Worksite health promotion involves a broad mix of stakeholders. Each stakeholder is involved in worksite health promotion in a different way. The Dutch situation is largely determined by the privatisation of the sickness absenteeism costs, competition and regulatory processes in the health care sector and the future shrinking size of the work force [15]. Also outside the Netherlands, worksite health promotion is a multidisciplinary domain, involving a broad range of stakeholders, resulting in ‘a complex, poly-vocal approach’ ([6], p77).

Meershoek, Bartholomée and Horstman [15] identified - next to the employer and employee - the government, occupational physicians, insurance companies, research and knowledge institutes, intervention providers and labour unions to be involved as stakeholders in worksite health promotion in the Netherlands. Each stakeholder is involved in worksite health promotion in a different way. For example, employers can have several reasons to promote their employees’ health, such as cost-saving aspects (e.g. reducing sickness absenteeism), sustainable employability in the light of the ageing workforce, and good employment practice for company image building. With respect to the intervention providers, they commercially offer interventions and activities to employers and thereby derive their reason of existence. The employees are the target group of worksite health promotion and supposed to participate in the intervention or services on offer. Although they are the target group, they generally lack voice in worksite health promotion [15]. Because these stakeholders are involved in different ways, they bring their own views and considerations to the matter of worksite health promotion.

### Objective

Although worksite health promotion is becoming more common, to date, empirical research on ethical aspects of worksite health promotion is scarce. Therefore, this paper explores the views of several stakeholders involved in worksite health promotion. It furthermore describes the ethical considerations that result from differences between these views.

## Methods

### Study design

To explore the views of stakeholders involved in worksite health promotion and to describe the ethical considerations that result from differences between these views, eight focus group discussions were organised (one for each stakeholder group). Focus group discussions are semi-structured discussions of 4 – 12 participants. The focus groups were moderated by the first author. Participants answered individually to the questions of the moderator, but were encouraged to talk and interact with each other, as group interaction encourages participants to explore shared and individual views [16,17]. This study was part of a large research project called ‘Vitality In Practice’, of which design and procedures have been approved by the medical ethics committee of the VU University Medical Center. This part of the research project did not need specific ethical approval. This qualitative study adheres to the RATS guidelines for reporting qualitative studies (see Additional file 1 for the RATS checklist).

### Setting

The focus group discussions took place in central location, with an acceptable travelling distance for all participants throughout the Netherlands. Participants received no financial remuneration for their participation, but did receive a small present as a token of appreciation. Travelling expenses were remunerated. In addition to the focus group discussions, face-to-face interviews were held, when participants were not able to join the focus group discussions.

### Participant selection

The focus groups were organised per stakeholder, instead of mixing the different stakeholders into groups, in order to avoid social desirable answers, influenced by possibly perceived hierarchy. Participants were sampled by means of stratified purposive sampling [18], which illustrates characteristics of particular subgroups of interest as this facilitates comparisons. Employees [EE] (n = 6) and employers [ER] (n = 4) were selected from large (>250 employees) and smaller organisations (<25 employees) and from two different sectors (industry and service). To represent the government [GO] (n = 3), policy advisors from both of the ministry of Health, Welfare and Sports and of the ministry of Social Affairs and Employment were selected. Research and knowledge institutes [RKI] (n = 5) were represented by senior and junior researchers active in the field of worksite health promotion of different institutes in the Netherlands. From different health insurance companies and income insurance companies [IC], staff officers and policy advisors (n = 4) were selected. Occupational physicians [OP] (n = 9) who worked independently, who worked in the same organisation as their clients (the employees) and who worked in an independent occupational health service were selected. Providers [PR] (n = 6) of different types of worksite health promotion activities were selected, such as activities aimed at improving lifestyle (physical activity, diet, smoking, etc.), activities aimed at improving mental health, and (integral) health management programs. Furthermore, representatives of labour unions [LU] (n = 7) of different sectors, such as blue and white collar, and profit and non-profit were selected.

Six of the eight focus groups consisted of four to nine participants. One focus group (insurance companies) consisted of only two participants as the other invitees reported to be sick at the moment of the focus group discussion. This focus group discussion was supplemented with two additional face-to-face interviews. One focus group (government) consisted of three participants, as one participant reported to have last minute other obligations. The three participants were all experts and the focus group was considered information rich, so that no additional face to face interviews were conducted for this stakeholder group. All participants signed informed consent before starting the interview.

### Data collection

The data were collected between November 2011 and February 2013. The focus groups were moderated by the first author [JvB]. The focus group discussion started with a short introduction of the study and signing informed consent forms. Next, the participants were asked to introduce themselves and their relation to worksite health promotion. All focus group discussions and additional face-to-face interviews were audio taped and transcribed verbatim. In addition, field notes were taken by a research assistant. Duration of the focus groups was about one hour and a half. The interview guide consisted of the following topics: 1) view on occupational health, 2a) view on worksite health promotion, 2b) view on stakeholders’ own role in worksite health promotion, and 2c) view on other stakeholders’ roles in worksite health promotion. The interview guide was pilot tested in a focus group discussion with master students in Health Sciences. For the additional face-to-face interviews, the same interview guide was used.

### Data analysis

A content analysis was performed, according to the constant comparison method [19]. With this method, themes in views emerged inductively from the transcripts and dilemmas based on differences between views emerged deductively through constant comparison. The first author [JvB] analysed all data. The first step was ‘open coding’; relevant passages were selected and coded, with often descriptions used by the participants (i.e. ‘descriptive codes’) [20]. Next, emerging and overarching themes (i.e. ‘analytic codes’) were identified among the codes, which best characterised the data collected. Throughout the analysis process, the codes and themes were constantly compared to the rest of the data. The research team consisted of several investigators [JvB, AM, RJ, CB, KP, AvdB], who all reflected on the research process (investigator triangulation) and reviewed and explored scientific and organizational aspects of this study during meetings (peer debriefing). Data analysis performed by the first author [JvB] was discussed with the second author [AM], to warrant accuracy (confirmability).

## Results

In our analysis, we distinguished the following themes; the definition of occupational health, occupational health risk factors, worksite health promotion, and responsibility.

### Definition of occupational health

In defining occupational health, all participants agreed that what is at stake if we talk about occupational health is that employees are able to do their job. They emphasised that this does not imply that people can not have health problems:

‘Occupational health is that you are physically able to perform the activities at work that you should do and that you do not experience any difficulties because of your health in undertaking activities.’ [EE1]

‘Occupational health is naturally not purely the absence of disease or defects. Someone can have one leg and still feel healthy and function well with only that one leg. To me, it means that there are no limitations that hinder functioning.’ [PR2]

### Risk factors for occupational health

Although the stakeholders all agreed on what occupational health is, they differed in what they considered to be important risk factors to occupational health. There was a dichotomy in types of risk factors: job-related and employee-related. These risk factors were noted by all stakeholders, but the importance they ascribed to these two categories differed.

Employees considered the job itself and working conditions as important risk factors for occupational health.

‘Because we work in a small company, well we have to produce a lot, and well that means that there is a lot of work that has to be done, and we are actually with too few people for the amount of work we have. High work pressure. And that is detrimental for your health.’ [EE6]

‘Well on some train routes, then you have to walk through the train to check train tickets, but well if you are on that route all day long, your knees are really sore at night. That is really miserable.’ [EE2]

Labour unions indicated that, to them, working conditions, such as shift work (nightshifts), aggression (social safety), indoor climate and air quality, are the most important risk factors to occupational health, because these are related to the worksite context.

‘Other aspects such as a healthy diet, enough physical activity, those are not specific only for occupational health, but for health in general. That is important for every citizen, not only for employees. Working conditions characterise occupational health.’ [LU6]

Occupational physicians also indicated the importance of work characteristics and working conditions for occupational health and put them first.

‘It should be the worksite first, that should be healthy, and you should not make an employee with a high workload work even harder.’ [OP8]

Yet, they felt working conditions are not as a big a risk as they once were.

‘Of course we want it [occupational health] to be better and better, but if we compare it to forty years ago, the working conditions are not merely as hard now as they were back then.’ [OP2]

Consequently, they also considered other risk factors to occupational health, such as mental health problems and sustainable employability.

‘Next to the physical things, there is also the mental side, that is just as important. If you look at why employees retire early, it is because they do not feel like it anymore, they are done with it. And not because they are not physically able anymore, but they are mentally worn out.’ [OP6]

The employers also considered some aspects of work and working conditions as a risk for occupational health, such as unsafe working conditions, and calamities, but also considered employee related issues such as lifestyle behaviours to be important risk factors.

‘An employee comes here with his capacities. The employee hires his capacities to us and if those capacities become 50% less because of the employee’s lifestyle, well then we have a problem with that as employer.’ [ER1]

Lifestyle behaviour was a broad definition in the opinion of the employers. It comprised dietary behaviour and physical activity, in a general sense, but for some employers also on a more detailed level, such as the choice of sports in case of sports injuries and leisure activities, such as going out drinking alcohol or using drugs. When it may influence productivity, it can be considered a matter of the employer. The employers argued that this is implied in the labour contract.

‘When you sign a contract, and you start working at our company, then you sign to do your work in optimal health and vitality. That’s what you get paid for. […] No, that is not literally mentioned of course.’ [ER1]

‘We do not accept that someone comes to work, bouncing under the influence of drugs […] and of course we do accept that someone plays soccer in his spare time, but if that someone gets a soccer injury for the third time, well then it is time to address that. Like: say, is playing soccer really the sport for you? […] When that someone persists on playing soccer and getting injured, and each time you can not work for three to four weeks and work performance suffers from it. Well then, eventually, it is time to say goodbye to that employee.’ [ER2]

Other stakeholders [RKI, GO, PR, IC] also noticed both job-related and employee-related risk factors, but they approach these risk factors more theoretically and impersonally, using terms as ‘environment’ and ‘behavioural determinants’. Health risk factors, which are explicit and ‘experienced’ in daily life by employers and definitely by employees, become abstract risk factors in this theoretical vocabulary. In addition, the government also considers another category of risk or difficulty in occupational health; namely the socio-economic status (SES) of the employee, as for the lower SES employees ‘their view on health and life experiences do not match our approach of occupational health’ [GO2]. In other words, according to this respondent, the socio-economic status influences the perceptions on health, making them less receptive for healthy lifestyle interventions. In explaining the influence of lower SES this way, the respondent reduces the structural factor of SES in a lifestyle issue.

### Worksite health promotion

The differences in what stakeholders considered to be important risk factors for occupational health, translated into what they consider to be potentially effective worksite health promotion activities. In the view of employees, examples of worksite health promotion are: ‘getting time off when it is convenient for me’ [EE1; EE5; EE6] or ‘vacation when I need it’ [EE3], design of a healthy working environment (for instance ‘the dead man’s switch in locomotives’ [EE3] or ‘working secured inside a wind mill’ [EE2], management’s planning of personnel to fulfil tasks [EE5; EE6] and mental guidance after disasters or other calamities [EE3; EE4]. The relationship between time off and occupational health was emphasised by employees. Labour unions considered ‘dialogue’ and ‘sustainable labour relationships’ important strategies to tackle job-related occupational health risk factors.

Other stakeholders primarily report strategies of worksite health promotion, aimed at employee related occupational health risk factors, for example: ‘inform and facilitate informed choice’ [OP], ‘seduce’ [OP], ‘goal setting’ [OP], ‘integrated health management’ [PR], ‘sustainable employability and vitality interventions’ [RKI], ‘vitality check’ [ER], ‘in-company fitness or fitness with reduced fee’ [ER; PR] and ‘environmental change’ [GO]. In addition, also worksite health promotion activities in work processes are mentioned, such as ‘rotation of tasks’ [OP] ‘efficiency of work processes’ [PR], ‘training in lifting techniques’ [PR]. Employees report that for instance fitness with a reduced fee are considered ‘a nice extra feature’ [EE3; EE5], but to their opinion does not tackle major issues in occupational health. The labour unions state that worksite health promotion activities as it is defined by the other stakeholders is “the icing on the cake” [LU4]. The cake represents the working conditions, and the icing represents in-company fitness and other worksite health promotion activities. “First the cake should be ready. Then it is time to add the icing” [LU3].

Some stakeholders, who are not directly involved in the worksite, give examples of worksite health promotion activities on a more abstract theoretical level, with terms as ‘evidence-based interventions’ [RKI; IC], ‘behavioural change’ [RKI;GO], ‘physical and social environment’ [RKI;GO; PR] and ‘effects’ [RKI; IC; GO; PR]. Those stakeholders link worksite health promotion activities to outcomes such as ‘productivity’ [PR; RKI], ‘burden of losses control’ [IC]. Again, in this vocabulary worksite health promotion is abstracted from every day experiences.

### Responsibility

‘Employees’ own responsibility’. Employees own responsibility for occupational health is key to all stakeholders, but how they interpret ‘responsibility’ differs between stakeholders. In employees, two elements can be distinguished in their view on their own responsibility for lifestyle. Firstly, lifestyle to them is “very personal”, which means that they do not owe any justification to employers for their lifestyle. To them, ‘their own responsibility’ equals *autonomy.* Yet, this notion is also differentiated by employees when work performance and safety are affected by lifestyle. They indicate that employees should ‘take responsibility’ for lifestyle behaviours when the results of these behaviours interfere with their functioning at work (‘if you weigh 130 kilos and you have to climb up and down ladders for your job, then you should do something about that’ [EE2]) or with safety (“Colleagues of mine had to work in Groningen for a couple of days and they stayed in a hotel. They drank so much that they weren’t able to do a responsible job in the morning” [EE1]).

Secondly, although they feel that lifestyle is their own free choice, they also think that the choices they make are influenced by other factors, such as work. Working conditions, for instance working schedules and night shifts force them into certain lifestyle choices. In addition, they consider for instance (genetic) ‘predisposition’ or “disease” to play a role for body weight, and personal preference and enjoyability to play a role in case of sports (‘Not everybody like sports’ [EE3]). They therefore feel that that employers can not ‘demand’ from employees to do something about lifestyle, employers can only ‘guide’ employees in these matters, as ‘not everyone can help it’ [EE3].

Regarding lifestyle, there is one aspect that they consider to be their responsibility, namely to be well rested for work (“take enough rest”), in order to be able to perform. Mental health, another aspect of occupational health, is considered to be more of a responsibility of the employer (“I think responsibility for mental health is seventy percent employer and thirty percent employee” [EE4]), because work pressure as applied by the employer is seen as a considerable risk factor for mental health problems.

Employers consider lifestyle to be the responsibility of the employee; ‘All those BRAVO (i.e. lifestyle behaviour) themes, that is 100% responsibility of the employee’ [ER2]) and they consider it a *duty* of the employee to ‘take his responsibility’. From the perspective that *responsibility* equals *duty*, ‘responsibility’ is also a target to address the employee. In other words; to the opinion of the employer, employees can be held accountable for their lifestyle behaviour and owe justification to the employer.

‘We have a policy on employability. That assumes of course the responsibilities of the employer, but certainly also of the employee. We do expect of the employees to do everything they can in order to be as employable as possible.’ [ER3]

Employers’ responsibility. Stakeholders generally consider it the responsibility of the employer to provide a healthy working environment, as they are required by law.

‘The employer should take care of a healthy working environment. According to the law, you should make sure that it is safe to work. What you do on top of that, is extra.’ [ER1]

As mentioned above, employees consider mental health a shared responsibility, with more responsibility of the employer than of the employee (see employees’ responsibility). Employers feel responsible for preventing mental health problems;

‘It is not so much the fact that employees become mentally ill, but the fact that we did not see it coming.’ [ER2].

Employers of large organisations have more attention for worksite health promotion than employers of smaller organisations. Insurance companies notice that employers of smaller organisations do often not have knowledge on human resource management or health management and their aims can be considered short-term. Their health aims often are more curative than preventive. Employers of larger organisations often issue their own policies, and have more often a long-term vision on occupational health. In larger organisations, preventive measures are more incorporated.

‘Smaller companies, we see that in our research, they focus much more on the short-term. They really do not engage in long-term health management, but they want to keep their personnel working. They want fast solutions, it is all ad-hoc. Smaller companies benefit from low-hanging fruit, from quick-wins, whereas the larger companies, they have their own policy. Often they also do the acquisition of care themselves. Larger companies are a) more interested in knowledge transfer such as health management conferences and b) they see us as sort of provider.’ [IC4]

Other stakeholders responsibilities. There seems to be a discrepancy between what stakeholders do (or think they do) in worksite health promotion, and what they potentially could do, in the eyes of other stakeholders. This is particularly the case for occupational physicians and labour unions.

Occupational physicians mainly deal with worksite health promotion, as part of sickness absenteeism attendance, which is mainly ‘at the back end of the channel’ [OP8]. Many stakeholders, such as government, insurance companies, and research and knowledge institutes, think occupational physicians have too little focus on health promotion and prevention, and see opportunities for occupational physicians to play a larger role.

‘I think occupational physicians, at least at this moment, play a marginal role in worksite health promotion. Because they are educated to guide sickness absence and to re-integrate employees back to work, while worksite health promotion is behavioural and environmental change. They are not trained and educated to do that, and they are not taken seriously by employers or human resource management as a party in these matters.’ [RKI2]

‘When the commercial occupational health and safety services were founded, we saw that they all aimed to reduce sickness absenteeism. It resulted in, well, halving the numbers of sickness absenteeism. Thus, the occupational health and safety services have done an excellent job. However, they have not succeeded in positioning themselves as a service provider in the area of sustainable employability and worksite health promotion. Everyone is undertaking attempts but what we see when we look at the numbers, is that turnover is declining year after year. None of the services have been able to follow the trends.’ [IC2]

Labour unions see ‘negotiating’ and ‘mediating’ on different levels (sector, collectively, individually) between employers and employees as their responsibility in worksite health promotion. However, both employers and employees mention that worksite health promotion is not a topic that is normally addressed with labour unions, although it seems to differ between different unions. Also other stakeholders, such as research and knowledge institutes, consider that labour unions could do more as ‘interlocutor’ for worksite health promotion.

‘I’m a member of the XXX union, and we never really talk about worksite health promotion, no.’ [EE4]

‘I think that the unions could potentially play a very important role in worksite health promotion, and you see that there is a shift going on. Until now, they were the big absentee in these matters. Why? Because they held on to the traditional occupational health risk factors, they argued that employers should focus on that first.’ [RKI2]

### Ambivalent relationships between stakeholders

Stakeholders have different perspectives on reciprocal responsibilities, which in practice leads to ambivalent relationships between stakeholders. Ambivalence in its turn may contribute to distrust. According to the stakeholders, differences in views on worksite health promotion lead to an ambivalent relationship between employer and employee in the practical context of the worksite, and in addition, employees indicate they do not trust other stakeholders who are –in their view- on the side of the employer, such as the occupational physician.

‘We have a special functionary for this [human resources management], but well, yes he is very close to the management. It is more of a staff position. So, well, yes, how trustworthy is such a person? And we also have an occupational physician, but how trustworthy is he in this kind of matter?’ [EE5]

The employers reasons to engage in worksite health promotion were questioned, but also the type of worksite health promotion was questioned.

‘I often find the employer very short-sighted. They often only look at their own interests. For instance, our manager is a very fanatic sportsman, a real health freak. So he thinks that other people will do it as well when he says so, but that is not the way it works of course.’ [EE3] ‘No, not everyone likes sports.’ [EE1]

Employers indicated they notice the ambivalence in the relationship with their employees. They think there are no grounds for distrust against them.

‘It startled me that there is such a distrust. I think, we surely try to communicate openly and transparently which each other, and for heaven sake, why should one possibly think it [information about health] would be used against you? So, that has surprised me and then raises the question, yes well how far should you go in interfering in the personal life of your personnel.’ [ER2]

The ambivalent employer-employee relationship is signalised by the labour unions. According to the labour unions, ‘employers tend to individualise the problem, and lack a collective, integrated approach’, although it is seen as ‘a collective matter’ by the labour unions, and ‘employees resist against such an individualised approach’ [LU5].

In addition, there is also ambivalent relationship between employers and labour unions.

‘I recognise the ‘dugging heels in the sand’ [of employees] at your company, so to say ‘what business is that of yours’?’ [ER2] ‘Yes, certainly in the beginning yes. And it is on all levels, and the labour unions do not help, because they are very suspicious in these matters [worksite health promotion].’ [ER1]

‘Employers expect healthy lifestyle of employees and expect help from us [LU], but this clashes as we feel employers should take care of working conditions first.’ [LU7]

Ambivalent relationships are also signalised by the research and knowledge institutes.

‘The employee often says: the occupational physician is hired by the employer. […] I have had interviews with employees and they said that they did not want the occupational physician to know about it [health issues]. There really is such a distrust.’ [RKI4]

## Discussion

In this paper, we aimed to explore the views of stakeholders involved in worksite health promotion and their ethical considerations by conducting focus group discussions, and analysing these through constant comparison.

To date, and to the best of our knowledge, this is the first study exploring the views of stakeholders of worksite health promotion to identify ethical considerations. Previous studies have investigated ethical considerations of prevention or health promotion in general (e.g. [13]. However, the stakeholders, and particularly the dependency relationship between employer and employee leads to the fact that worksite health promotion is not well comparable to health promotion in general. Robroek and colleagues [3] established that ethical considerations can play a role in employees’ decision whether or not to participate in worksite health promotion, but they did not consider the views of other stakeholders. With providing insight in stakeholders’ perceptions, our study gives a more empirical exemplification of the theoretical reflections on ethical aspects of worksite health promotion other studies give. Another strength of our study lies in the richness of experiences as expressed by our participants. Working with a limited number of participants enabled us to examine their responses in depth. Due to limited number of respondents, we supposedly have not covered all potential ethical considerations. Yet, we did reveal relevant ones. A limitation of this study is that we may have not interviewed all parties involved in worksite health promotion. For example, the occupational health care system in the Netherlands comprises more health professionals than occupational physicians.

Although we investigated worksite health promotion only in the Dutch context, the multi-disciplinarity of the field of worksite health promotion involving several stakeholders, is more universal [6,21,22]. We can therefore assume that similar ethical considerations exist in other countries.

Our analyses showed that although the definition of occupational health is the same for all stakeholders, namely ‘being able to perform your job’, there seems to be some difference in the views on risk factors for occupational health. This difference can be translated into a dichotomy of job-related and employee-related health risk factors. According to the employees, occupational health risk factors are prevailingly job-related. Labour unions agree with them, but other stakeholders, including the employer, particularly see employee-related risk factors, such as lifestyle behaviours. The differences in definition of occupational health risk factors translates roughly into a comparable dichotomy of job-related and employee-related worksite health promotion activities. This difference in conceptualisation indicates that worksite health promotion is generally not employee-driven. This finding is supported by Meershoek et al. [15] as they concluded that employees lack voice in worksite health promotion.

The difference in conceptualisation of occupational health risk factors and worksite health promotion also resonates in the way stakeholders understand ‘responsibility’. Even though all stakeholders agree on whose responsibility healthy behaviour is, namely that of the employee, the meaning of ‘responsibility’ differs between employees, and employers. For employees, responsibility means *autonomy*, while for employers and other stakeholders, responsibility equals *duty*. Previous studies have found autonomy to play a role in ethical considerations of worksite health promotion along the lines of voluntariness to participate in a worksite health promotion activity [7,9,13,14]. We can wonder whether this emphasis on voluntariness does not become an empty letter, if responsibility is understood as a duty. The conceptualisation of responsibility as duty, furthermore enlarges the risk of ‘blaming the victim’ [6,7,9]. Responsibility conceptualised as duty is based on the assumption that occupational health is individual and amendable, thereby ignoring the multifactorial composition of occupational health.

The difference in conceptualisation of responsibility may contribute to mutual distrust between stakeholders. The employers’ expectation that signing a labour contract implies that employees have a duty to care for their health can namely be seen as a so-called ‘psychological contract’ [23]. This demands a match in reciprocal beliefs about what the other parties’ responsibilities and duties are. Mismatches in reciprocal beliefs, especially on responsibility, may contribute to a mutual distrust, which we found between employer and employee, between employer and labour unions, and between employee and occupational physician. The perception of conflicting loyalties [6,7] adds to ambivalence in relationships. This is a major issue, as the employer-employee relationship is a key element in worksite health promotion. This is even more the case in smaller organisations, where due to the shorter lines, the employer-employee relationship is of even greater influence.

### Implications for research and practice

Developing, implementing and evaluating worksite health promotion requires dealing with all stakeholders involved, and consequently with their views and ethical considerations. Assuming that professionals in the field of worksite health promotion are usually not profoundly trained in ethics, we recommend for professionals in both research and practice to consider the following implications of our study.

Firstly, all stakeholders, including employees, should be given a voice in developing, implementing and evaluating worksite health promotion. Secondly, since stakeholders agree on lifestyle being the responsibility of the employee, but disagree on what this responsibility means (duty versus autonomy), it is of utmost importance to examine the discourse of your stakeholders. This way, ambivalence in relationships between stakeholders could be prevented.

## Conclusion

This difference in conceptualisation of occupational health risk factors and worksite health promotion resonates in the way stakeholders understand ‘responsibility’ for lifestyle behaviour. Even though all stakeholders agree on whose responsibility lifestyle behaviour is, namely that of the employee, the meaning of ‘responsibility’ differs between employees, and employers. For employees, responsibility means autonomy, while for employers and other stakeholders, responsibility equals duty. This difference may in turn contribute to ambivalent relationships between stakeholders.

## Competing interests

The authors declare that they have no competing interests.

## Authors’ contributions

All authors contributed to the conceptual design of the study and intellectual input into the design of this paper. JvB performed data collection, and analysed the data together with AM. JvB drafted the manuscript. MJPAJ, CRLB KIP, and AJvdB provided support during the data collection and reflected on the research process. All authors contributed to the further writing of the manuscript. All authors read and approved the final manuscript.

## Pre-publication history

The pre-publication history for this paper can be accessed here:

http://www.biomedcentral.com/1471-2458/14/458/prepub

## Supplementary Material

Additional file 1 RATS checklist. Click here for file
